# Practical guidance for managing patients with moderate-to-severe ulcerative colitis using small molecule therapies

**DOI:** 10.1093/jcag/gwae013

**Published:** 2024-05-15

**Authors:** Vipul Jairath, Waqqas Afif, Brian Bressler, Janet E Pope, Daniel Selchen, Laura E Targownik, Remo Panaccione

**Affiliations:** Division of Gastroenterology, Western University, St. Joseph’s Health Care, London, ON, Canada; Division of Gastroenterology and Hepatology, McGill University, Montreal General Hospital, Montreal, QC H3G 1A4, Canada; Division of Gastroenterology, University of British Columbia, Vancouver, BC V5Z 1M9, Canada; Division of Rheumatology, St Joseph’s Hospital, Western University, London, ON N6A 4V2, Canada; Division of Neurology, Barlo Multiple Sclerosis Centre, Department of Medicine, St. Michael’s Hospital, University of Toronto, Toronto, ON M5B 1W8, Canada; Division of Gastroenterology and Hepatology, Department of Medicine, University of Toronto, Toronto, ON M5S 3H2, Canada; Mount Sinai Hospital IBD Centre, Division of Gastroenterology, Mount Sinai Hospital, Toronto, ON M5G 1X5, Canada; Division of Gastroenterology and Hepatology, Department of Medicine, University of Calgary, Calgary, AB T2N 4Z6, Canada

## Abstract

Ulcerative colitis (UC) is a severe and debilitating illness that affects the quality of life and physical health of many Canadians. Given the dynamic and progressive nature of the disease, advanced therapies are required to support its long-term management. The emergence of small molecule therapies offers novel treatment options that target mechanisms central to the immunopathology of UC. Sphingosine-1-phosphate (S1P) receptor modulators and Janus-activated kinase inhibitors are 2 classes of therapies that target unique pathways to attenuate inflammation and modulate the immune response characteristic of UC. This review aims to provide practical guidance on how these therapeutic options can best be used to optimize treatment management and highlight the emerging role of small molecule therapies as a treatment strategy for UC.

## Introduction

Ulcerative colitis (UC) is a severe, debilitating, life-long illness characterized by inflammation of the colorectal mucosa, which occurs in a relapsing and remitting manner.^1^ Of the 322 600 Canadians living with inflammatory bowel disease (IBD), approximately 160 000 currently live with UC or unspecified IBD; approximately 6800 new cases are diagnosed each year.^2^ The prevalence of UC in Canada is increasing, and it is expected that the number of Canadians living with UC will increase from 260 per 100 000 in 2015 to 436 per 100 000 by 2030.^3,4^ The growing rate of UC in Canada, coupled with the chronic nature of the condition, places a significant burden on the healthcare system and carries a substantial financial cost. The total healthcare costs associated with the burden of IBD in Canada, including UC, are estimated to be $1.3 billion/year, attributed primarily to prescription drugs and hospitalizations, with an additional $1.26 billion owed to lost wages, absenteeism, and out of pocket expenses.^5^ As such, improving the treatment options for patients with UC is essential.

Treatment of UC can be challenging due to the chronicity and variability of the disease course, both among and within patients. This manuscript was developed to be a resource providing practical guidance for managing patients with UC on small molecule therapies. This work was developed from a series of meetings where Canadian physicians, with clinical experience in managing UC and the use of small molecule therapies, provided expert opinions and insights on the topic, supported by available published data.

## Treatment of ulcerative colitis

The goal of UC treatment is to induce and maintain remission, while minimizing exposure to corticosteroids, and ultimately avoiding surgery.^6^ Given the dynamic nature of this condition, patients with UC often require treatment modifications due to changes in treatment effectiveness, safety, and tolerability.^7^ Patients are often switched to a therapy with a new mechanism of action upon failure of a given treatment to best manage disease outcomes and achieve treatment goals. The emergence of new treatment strategies designed to target novel mechanisms of action is advantageous in providing alternative therapies to support ongoing and ever-changing patient needs.^8^

The recommended first-line of therapy for mild-to-moderate UC is oral or rectal 5-aminosalicylate (5-ASA), with oral corticosteroids as second-line therapy.^6^ For moderate-to-severe UC, current guidelines suggest that systemic corticosteroids,^6^ alone or in combination with oral mesalamine,^9^ should be considered as first-line therapy. However, corticosteroids are not indicated for maintenance of remission due to side effects associated with prolonged use, including cardiovascular events, mood disturbances, hyperglycaemia, avascular necrosis, and bone density loss.^10,11^ Thiopurine monotherapy is not recommended for induction of remission^12^ but is an effective corticosteroid-sparing treatment for maintenance of remission in UC.^13–15^ Biologic therapies may also be used to treat UC, including monoclonal antibody therapies targeting tumour necrosis factor-alpha (TNF-α), α_4_β_7_ integrin, interleukin-12 and interleukin-23 (IL-12/IL-23), or IL-23. Thiopurines may be used in combination with anti-TNF therapies to enhance the efficacy of treatment and reduce the risk of developing antibodies against the biologic.^16^ However, half of patients on biologic therapies will develop primary failure or eventually lose response to these treatments,^17,18^ requiring a change in therapy or surgical management. The development and implementation of novel lines of therapy for the management of UC is a high priority.

## Advanced small molecule therapies in ulcerative colitis

**Figure 1.**
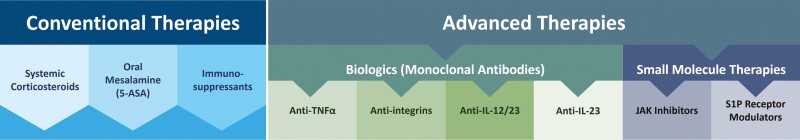
Advanced small molecule therapies that target novel mechanisms of action are emerging as an effective treatment for moderate-to-severe ulcerative colitis.

Advanced small molecule therapies, such as sphingosine-1-phosphate (S1P) receptor modulators and Janus-activated kinase (JAK) inhibitors, are among the emerging UC treatment options designed to provide patients with alternatives to support long-term treatment goals (Figure 1). These treatment options have novel mechanisms of action for patients to manage symptoms effectively and relatively safely. Advanced small molecule therapies generally have a lower risk of immunogenicity compared to biologics, which can lead to sustained efficacy and a more consistent treatment response. Further, the short half-lives and flexibility in dosing allow for individualized dosing, timely adjustments, and mitigation of side effects.

Current treatment guidelines in Canada have yet to incorporate specific recommendations for the use of these small molecule therapies for the treatment of UC; however, the Selecting Therapeutic Targets in Inflammatory Bowel Disease (STRIDE)-II guidelines provide monitoring recommendations, which may influence the selection of small molecule treatment in UC.^19^ While advanced small molecule therapies are a recent innovation in the treatment of UC, they have been used extensively to treat autoimmune diseases, such as rheumatoid arthritis, psoriatic arthritis, axial spondyloarthritis, atopic dermatitis, and multiple sclerosis.^20,21^ As such, there is a wide body of knowledge that already exists about these therapies, which may be applied to their use as a treatment for UC.

## S1p receptor modulators

Sphingosine-1-phosphate receptor modulators target a unique series of intracellular signalling cascades to modify lymphocyte trafficking, cell proliferation, migration, and apoptosis.^22^ Four S1P receptor modulators have been approved by Health Canada for treatment of multiple sclerosis (fingolimod, ozanimod, siponimod, and ponesimod).^23^ In 2022, ozanimod became the first S1P receptor modulator approved in Canada for the treatment of UC.^24^ Another S1P receptor modulator, etrasimod, was approved by Health Canada in 2024 for the treatment of UC.^25^ Ozanimod functions by binding to S1P1 and S1P5 receptor subtypes, which are important in signaling lymphocyte transfer out of lymph nodes to sites of injury. Etrasimod functions by selectively activating S1P receptor subtypes 1, 4, and 5.^26^ The blockade of S1P receptors prevents lymphocytes from being recruited into the colon, allowing the mucosa to heal.^27^

The double-blind placebo-controlled phase II TOUCHSTONE clinical trial (NCT01647516) examined 2 doses of ozanimod (0.5 mg and 1 mg PO OD) in patients with moderate-to-severe UC for up to 32 weeks (Table 1).^28^ Patients receiving the 1 mg dose had a higher rate of clinical remission after 8 weeks of treatment, compared with the placebo group. Clinical response and mucosal healing were improved with both doses.^28^ The multicentre, randomized, double-blind, placebo-controlled phase III True North trial (NCT02435992) examined the efficacy and safety of ozanimod in patients during induction and maintenance treatment (up to 52 weeks).^7^ The study found increased clinical remission (18.4% vs. 6.0%), clinical response (47.8% vs. 25.9%), endoscopic improvement (27.3% vs. 11.6%), and mucosal healing (12.6% vs. 3.7%) in patients after the 10-week ozanimod induction period. After the 52-week maintenance period, the ozanimod treatment group had higher levels of clinical remission (37.0% vs. 18.5%), clinical response (60.0% vs. 41.0%), endoscopic improvement (45.7% vs. 26.4%), maintenance of remission (51.9% vs. 29.3%), glucocorticosteroid-free remission (16.7% vs. 29.6%), mucosal healing (29.6% vs. 14.1%), and durable remission (17.8% vs. 9.7%), compared with placebo. Analysis of clinical remission during the induction period of ozanimod found a non-significant trend favouring anti-TNF-naïve patients (22.1%) over anti-TNF-experienced patients (10.0 %).^7^ This suggests that ozanimod may be effective when positioned after first-line conventional therapies and before biologics or positioned after biologics (e.g., in patients who lose response to biologics).

**Table 1.** Results of clinical trials of S1P receptor modulators in moderate-to-severe UC.

Trial	*N*	Comparators	Primary outcome	Relevant secondary outcomes
TOUCHSTONE (phase 2)^26^	197	Ozanimod 0.5 mg OD and 1.0 mg OD vs. placebo	Clinical remission at wk 8: 16% (1 mg) and 14% (0.5 mg) vs. 6% (*P* = 0.048 and 0.14, respectively)	Clinical remission at wk 32: 21% (1 mg) and 26% (0.5 mg) vs. 6% (*P* = 0.01 and 0.002, respectively)
True North (phase 3)^7^	457	Ozanimod 1 mg OD vs. placebo	Clinical remission at wk 52 (among those who responded at wk 10): 37.0% vs. 18.5% (*P* < 0.001)	Clinical response at wk 52 (among those who responded at wk 10): 60.0% vs. 41.0% (*P* < 0.001)
ELEVATE UC 12 (phase 3)^27^	354	Etrasimod 2 mg OD vs. placebo	Clinical remission at wk 12: 25% vs. 15% (*P* = 0.026)	Clinical response at wk 12: 62% vs. 41% (*P* = 0.0002)
ELEVATE UC 52 (phase 3)^27^	433	Etrasimod 2 mg OD vs. placebo	Clinical remission at wk 52: 32% vs. 7% (*P* = 0.0001)	Clinical response at wk 52: 62% vs. 34% (*P* < 0.0001)

Two randomized, double-blind, placebo-controlled trials (ELEVATE UC 52 and ELEVATE UC 12) examined efficacy and safety of maintenance and induction periods (NCT03945188 and NCT03996369, respectively) of etrasimod. Both trials found increased levels of clinical remission (UC 52: 28% vs. 8%; UC 12: 26% vs. 15%), endoscopic improvement (UC 52: 37% vs. 17%; UC 12: 33% vs. 19%), symptomatic remission (UC 52: 46% vs. 22%; UC 12: 48% vs. 29%), and histologic remission (UC 52: 23% vs. 6%; UC 12: 17% vs. 9%) during the induction periods, which were sustained during the maintenance period of the ELEVATE UC 52 trial (clinical remission: 33% vs. 8%; endoscopic improvement: 39% vs 13%; symptomatic remission: 44% vs. 19%; histologic remission: 27% vs. 10%). Subgroup analyses indicate treatment may be more effective in biologic-naïve patients than biologic-experienced patients.^26^ Similar to ozanimod, this suggests that etrasimod is effective when positioned after conventional therapies and before biologics, as well as if used after biologics.

Given the unique mechanism of action of S1P receptor modulators, this line of therapy presents a novel treatment strategy to support patients who have failed other therapies. Ongoing trials for S1P receptor modulators, as well as continued generation of real-world evidence will help to establish the role of S1P receptor modulators and confirm where this unique line of therapy will best be positioned in sequencing for Canadian patients living with moderate-to-severe UC.

While symptom improvement may be observed within 2 weeks of treatment with S1P receptor modulators,^29^ post hoc analyses from the True North trial found a delayed response of 5 weeks in approximately half of patients who did not respond within the 10-week induction period.^30^ Further, the proportion of patients with symptomatic remission plateaued around 16 weeks of treatment in the ELEVATE UC 52 trial.^26^ As such, patients should continue treatment for 8–16 weeks before considering withdrawal due to lack of response unless symptoms worsen during this time. Follow-ups should occur until the patient is established on their treatment, after which they can be individualized to each patient and their specific disease state.

## Managing adverse effects of s1p receptor modulators

**Figure 2.**
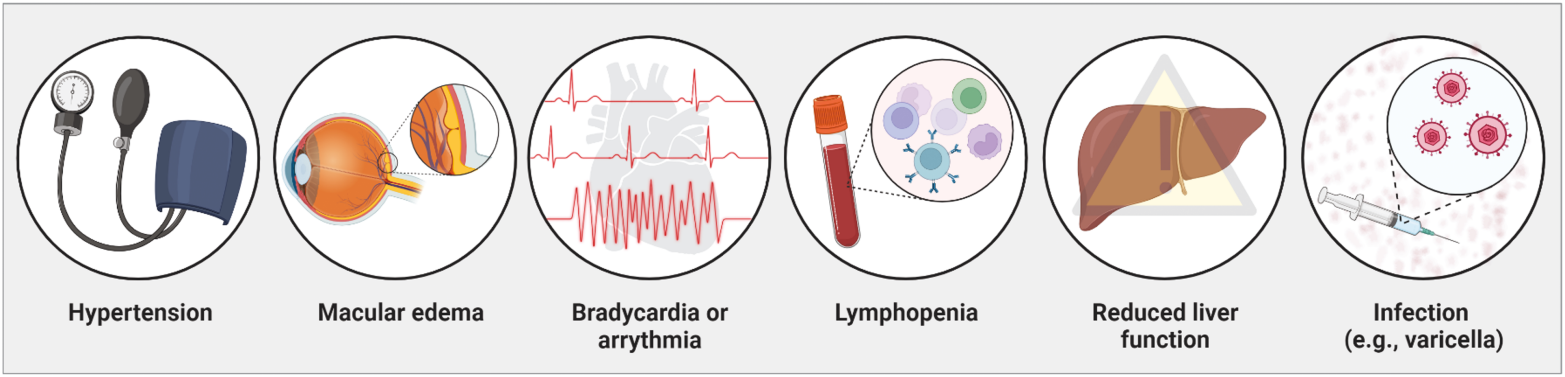
Side effects of S1P receptor modulators in moderate-to-severe ulcerative colitis. Side effects of S1P receptor modulators include, but are not limited to, hypertension, macular oedema, bradycardia or arrythmia, lymphopenia, reduced liver function, and development of shingles or varicella.

Monitoring for side effects and avoiding contraindications are key to providing optimal care for patients undergoing treatment for UC (Figure 2). While immediate side effects are unlikely to occur with S1P receptor modulators, there are some conditions that may develop, especially in patients with certain risk factors. People with borderline high blood pressure or hypertensive patients may experience an increase in blood pressure over the first few months of treatment, while normotensive patients may not see any effect.^31^ Patients should have their blood pressure measured regularly during treatment.^32^

Dose escalation is recommended to offset the threat of bradycardia or atrioventricular conduction delays, which can develop with treatment initiation of ozanimod.^24,33^ Patients should receive an electrocardiogram prior to initiating treatment of ozanimod to determine if there are any pre-existing conduction abnormalities.^24^ A referral to a cardiologist should be made for patients with certain pre-existing cardiac conditions: significant QT prolongation, ischaemic heart disease, heart failure, a history of cardiac arrest or myocardial infarction, cerebrovascular disease, uncontrolled hypertension, severe untreated sleep apnoea, a history of second-degree Mobitz type II or higher atrioventricular (AV) block, sick-sinus syndrome, sinoatrial heart block, a history of recurrent syncope or symptomatic bradycardia, or arrhythmias requiring treatment with Class Ia or Class III antiarrhythmic drugs.^24^

Patients with UC may be at an increased risk of shingles or varicella.^24^ In addition to knowing the patient’s varicella vaccination status, the Canadian Association of Gastroenterology (CAG) recommends offering the herpes zoster vaccine if the patient is currently unvaccinated against shingles and is aged 50 or older; the CAG also suggests offering the vaccine to patients under 50.^34^ Additionally, due to the immunosuppressive properties of S1P receptor modulators, which can reduce circulating lymphocyte counts (absolute lymphocyte counts of less than 200 cells/mm^3^ occurred in 1.1% of patients receiving ozanimod in the True North trial),^7^ physicians should obtain a complete blood count (including lymphocyte count), check human papilloma virus (HPV) vaccination status, and delay treatment initiation in patients with severe active infections.^24^ HPV screening is recommended for patients who have not been vaccinated against HPV, as treatment with S1P receptor modulators is associated with human papilloma virus infections, including papilloma, dysplasia, warts, and HPV-related cancer.^24^

Macular oedema may occur in 0.4%–1.2% of persons who are prescribed S1P receptor modulators, with increased risk in patients with a history of diabetes,^24,35^ a history of uveitis or macular oedema,^24^or age over 40 years.^36^ Persons with diabetes mellitus, uveitis, or a history of retinal disorders should undergo ophthalmologic screening within 4 months of starting treatment and throughout treatment.^24,37^ Persons using S1P receptor modulators who develop symptoms of macular oedema, including rapid loss of visual acuity, blurry vision, or visual field defects, should have prompt ophthalmologic examination to prevent permanent vision loss.^38^ Possible benefits and risks for the patient should be taken into consideration when determining how to proceed with minimizing the risk of vision loss; strategies may include treating the macular oedema or discontinuing S1P receptor modulator therapy.

Treatment with S1P receptor modulators is not recommended for patients with hepatic impairment, as the medication is hepatically cleared and may cause transaminitis.^24,39^ Other contraindications to ozanimod use include hypersensitivity to the drug; myocardial infarction, unstable angina, transient ischaemic attack, decompensated heart failure requiring hospitalization or Class III/IV heart failure within the previous 6 months; a history or presence of second-degree AV block Type II or third-degree AV block, sick-sinus syndrome, or sinoatrial block unless the patient has a functioning pacemaker; increased risk of opportunistic infections; severe active infections; known active malignancies (except localized basal cell carcinoma of the skin); pregnancy or childbearing potential not using effective contraception; and with concomitant use of MAO inhibitors.^24^ Breast-feeding patients should also not receive S1P receptor modulators.^24,40^

It should be noted that most side effects caused by S1P receptor modulators are not severe and can be managed through schedule change or using ancillary medications, such as anti-nausea agents to alleviate nausea or acetaminophen to control fever. Physicians may find it beneficial to use available resources during the induction period, such as patient support programs.

## JAK inhibitors

JAK inhibitors are another small molecule line of therapy for the treatment of moderate-to-severe UC. These therapies target the JAK family of intracellular tyrosine kinases, of which 4 different isoforms have been identified (JAK1, JAK2, JAK3, and tyk-2). JAK proteins are a critical component of the intracellular signaling cascade triggered by cytokines binding to external membrane-bound receptors, which mediate gene transcription characteristic of immune and inflammatory responses.^41^ Inhibition of JAK signaling prevents downstream activation of STAT proteins, in turn attenuating the nuclear transcription of inflammatory and immune-related signals.^9,10^

While several JAK inhibitors are approved by Health Canada for treatment of rheumatoid arthritis (baricitinib, tofacitinib, upadacitinib),^42^ tofacitinib and upadacitinib are currently the only JAK inhibitors indicated for treatment of UC. They are indicated for moderate-to-severe UC in patients who have displayed an inadequate response, loss of response, or intolerance to conventional therapies or a TNF-α inhibitor.^43–45^ Tofacitinib functions as an oral pan-JAK inhibitor with a preference for inhibition of JAK1 and JAK 3,^46^ while upadacitinib is a selective JAK1 inhibitor.^47^

Three phase III randomized, double-blind, placebo-controlled trials (OCTAVE Induction 1 [NCT01465763], OCTAVE Induction 2 [NCT01458951], and OCTAVE Sustain [NCT01458574]) examined the efficacy and safety of induction and maintenance therapy with tofacitinib in adults with moderate-to-severe UC (Table 2).^48^ Patients experienced improved clinical remission (Induction 1: 18.5% vs. 8.2%; Induction 2: 16.6% vs. 3.6%), mucosal healing (Induction 1: 31.3% vs. 15.6%; Induction 2: 28.4% vs. 11.6%), clinical response (Induction 1: 59.9% vs. 32.8%; Induction 2: 55.0% vs. 28.6%), and endoscopic remission (Induction 1: 6.7% vs. 1.6%; Induction 2: 7.0% vs. 1.8%) after 8 weeks of induction or 52 weeks of maintenance (clinical remission: 5 mg and 10 mg, 34.3% and 40.6% vs. 11.1%; mucosal healing: 5 mg and 10 mg, 37.4% and 45.7% vs. 13.1%; clinical response: 5 mg and 10 mg, 51.5% and 61.9% vs. 20.2%), compared with placebo. The proportion of patients with sustained and glucocorticosteroid-free remission, who were in remission at maintenance trial entry, was elevated after 52 weeks of tofacitinib treatment. Treatment efficacy appeared similar between anti-TNF-naïve and anti-TNF-experienced patients.^48^

**Table 2.** Results of clinical trials of JAK inhibitors in moderate-to-severe UC.

Trial	N	Comparators	Primary outcome	Relevant secondary outcomes
OCTAVE Induction 1 (phase 3)^47^	598	Tofacitinib 10 mg BID vs. placebo	Clinical remission at wk 8: 18.5% vs. 8.2% (*P* = 0.007)	Mucosal healing at wk 8: 31.3% vs. 15.6% (*P* < 0.001)
OCTAVE Induction 2 (phase 3)^47^	541	Tofacitinib 10 mg BID vs. placebo	Clinical remission at wk 8: 16.6% vs. 3.6% (*P* < 0.001)	Mucosal healing at wk 8: 28.4% and 11.6% (*P* < 0.001)
OCTAVE Sustain (phase 3)^47^	593 who responded at wk 12	Tofacitinib 5 or 10 mg BID vs. placebo	Clinical remission at wk 52 (among those who responded at wk 12): 34.3% (5 mg) and 40.6% (10 mg) vs. 11.1% (*P* < 0.001 for both comparisons)	Mucosal healing at wk 52 (among those who responded at wk 12): 37.4% (5 mg) and 45.7% (10 mg) vs. 13.1% (*P* < 0.001 for both comparisons)
U-ACHIEVE induction (phase 3)^48^	474	Upadacitinib 45 mg OD vs. placebo	Clinical remission at wk 8: 26% vs. 5% (*P* < 0.0001)	Mucosal healing at wk 8: 11% vs. 1% (*P* < 0.0001)
U-ACCOMPLISH (phase 3)^48^	522	Upadacitinib 45 mg OD vs. placebo	Clinical remission at wk 8: 34% vs. 4% (*P* < 0.0001)	Mucosal healing at wk 8: 11.3% vs. 2% (*P* < 0.0001)
U-ACHIEVE maintenance (phases 3)^48^	451 who responded at wk 8	Upadacitinib 15 mg OD and 30 mg OD vs. placebo	Clinical remission at wk 52 (among those who responded at wk 8): 42% (15 mg) and 52% (30 mg) vs. 12% (*P* < 0.0001 for both comparisons)	Mucosal healing at wk 52 (among those who responded at wk 8): 18% (15 mg) and 19% (30 mg) vs. 5% (*P* < 0.0001 for both comparisons)

A phase III, multicentre, randomized, double-blind, placebo-controlled study was comprised of 2 replicate induction studies (U-ACHIEVE induction (UC1) and U-ACCOMPLISH (UC2)) and one maintenance study (U-ACHIEVE maintenance (UC3)) examining the efficacy and safety of upadacitinib for 8 or 52 weeks (U-ACHIEVE: NCT02819635; U-ACCOMPLISH: NCT03653026).^49^ The patients receiving upadacitinib had significantly increased clinical remission at end point (UC1: 26% vs. 5%; UC2: 33% vs. 4%; UC3: 15 mg and 30 mg, 42% and 52% vs. 12%), demonstrating a positive efficacy for the treatment. A phase III clinical trial evaluating the long-term safety and efficacy of upadacitinib in adults with UC (NCT03006068), with a maintenance period of up to 288 weeks, is currently ongoing. Ongoing use of JAK inhibitors in patients, coupled with emerging data, will provide further insight into the use of JAK inhibitors after failure of first-line conventional therapies or biologics to manage moderate-to-severe UC.

As with S1P receptor modulators, JAK inhibitors should be used for 8–16 weeks before considering discontinuing due to lack of effect. Symptoms may be noticeably reduced within 3 days of treatment induction,^50^ but some patients may require an extended induction period (16 weeks) to achieve a clinical response.^51^ Follow-ups should occur until the patient is established and may be personalized to each patient and their current disease status.

## Managing adverse effects of jak inhibitors

**Figure 3.**
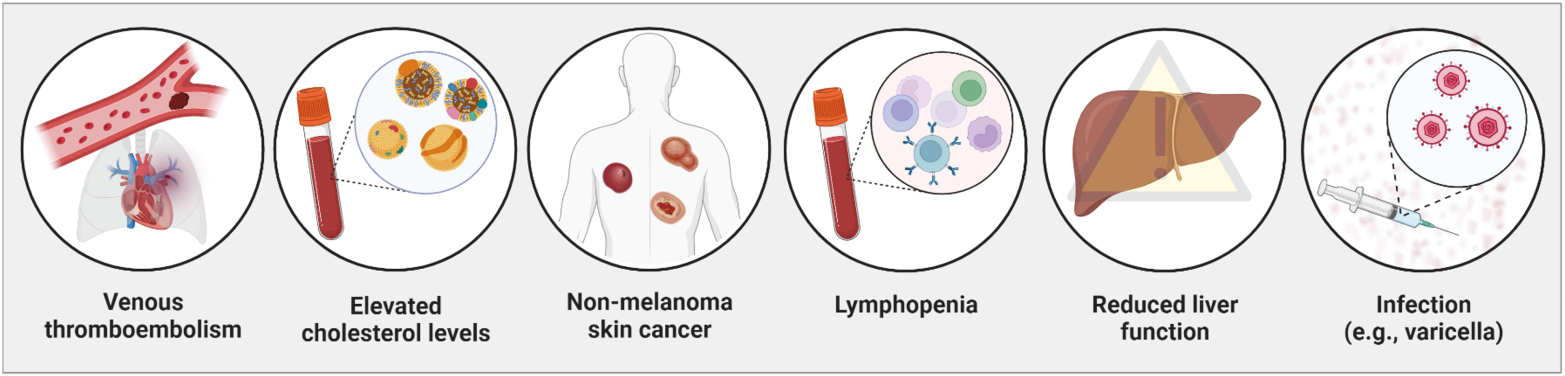
Side effects of JAK inhibitors in moderate-to-severe ulcerative colitis. JAK inhibitors may cause side effects including, but not limited to, venous thromboembolism, increased cholesterol levels, elevated risk of non-melanoma skin cancer, lymphopenia, reduced liver function, and increased risk of shingles or varicella.

Ensuring optimal care for patients treated with JAK inhibitors involves monitoring for side effects and avoiding contraindications (Figure 3). Physicians should obtain assessments of liver and renal function, as well as a complete blood count including lymphocyte and neutrophil counts, at baseline. Some JAK inhibitors are hepatically cleared, and liver function should be assessed periodically,^38,52^ with guidelines suggesting every 3 months.^53^ Monitoring lymphocyte counts in patients treated with JAK inhibitors is recommended every 3 months, as patients with an absolute lymphocyte count <500 cells/mm^3^ may be at a higher risk of infection.^37,52,54,55^

As with S1P receptor modulators, patients taking JAK inhibitors may be at an increased risk of shingles.^24^ Prior to initiating treatment, patients should be brought up to date with all immunizations, including prophylactic zoster vaccinations. Measurement of heart rate and blood pressure prior to treatment initiation is also recommended.^52^ JAK inhibitors should not be used during pregnancy or breast-feeding.^47,52^

The risk of venous thromboembolism (VTE) is known to be elevated among persons with IBD, especially those with severe disease requiring hospitalization. JAK inhibitors have been shown to increase the risk of VTE in patients with rheumatoid arthritis,^56–58^ and may not be an optimal choice in persons with VTE risk factors; however, given that the largest risk factor for VTE in UC is active disease, effective agents such as JAK inhibitors may be particularly appropriate in patients with severe disease.

JAK inhibitors can also alter levels of high-density lipoprotein and low-density lipoprotein and may increase the risk of cardiovascular events,^59,60^ although this is less likely in UC patients compared to rheumatoid arthritis patients, who tend to be older and have more comorbidities. Measurement of cholesterol levels may be done at onset of JAK inhibitor treatment, and then repeated 8 weeks after initiation. While most patients will not need lipid-lowering drugs, patients with abnormal levels of low-density lipoproteins or increased cardiovascular risk factors may require additional testing and/or consideration for lipid-lowering medications.^61^

JAK inhibitors also increase the risk of non-melanoma skin cancer, and the dose of tofacitinib should be reduced in patients with moderate-to-severe renal disease or moderate liver disease; JAK inhibitors are contraindicated in severe liver disease.^47,52,62^ Treatment benefits and side effects should be monitored closely to determine if dose modifications may be required.

## Strategies for treatment adherence

Strategies for monitoring adherence of small molecule therapies are limited, due to the simplicity of their delivery. Advanced small molecule therapies are taken orally, once or twice per day; if the patient misses a dose, they can continue as normal without double dosing the following day. It should be noted that if doses of S1P receptor modulators are missed for 2 weeks or more, the patient should be reinduced with a step-up course.

Small molecules are potent medications, despite their simplicity as an oral pill and physicians should emphasize the risks of self-dosing potent oral medications. Male patients with UC tend to be less adherent than female patients and other risk factors for non-adherence include younger age and single status.^63,64^ Understanding which patient characteristics are associated with increased likelihood of non-adherence may be beneficial when educating patients. Patients may be accustomed to the physician controlling treatment delivery, however, with small molecule therapies, the patient should understand the importance of treatment adherence to prevent exacerbation of symptoms, even during periods of remission.

## Conclusions

The emergence of small molecule therapies mirrors the growing understanding of the immunopathology of UC and provides novel treatment options for Canadian patients living with UC. As research continues to define the specific mechanisms underlying the symptoms of UC, advanced therapies can evolve to target these mechanisms more effectively. The continuing development of these small molecule therapies will allow agents to target specific isoforms of key molecules and receptors to elicit highly favourable and precise outcomes. Small molecule therapies will provide additional lines of therapy to support patients living with moderate-to-severe UC and offer renewed hope for the individuals who require additional lines of care.

## Supplementary Material

gwae013_suppl_Supplementary_Materials

## Data Availability

Data sharing is not applicable to this article, as no new data were created or analyzed in this manuscript.
